# John Lacy Firth

**Published:** 1943

**Authors:** 


### JOHN LACY FIRTH, M.D., M.S.(Lond.), F.R.C.S.

LACY FIRTH, a former president of the Society and Consulting Surgeon to the Bristol General Hospital—whose sudden death on April 26th, 1943, we deeply deplore—had for many years done valuable but unspectacular service to the Society and the *Journal*. In 1901 he began his work as reporter of the Society's Meetings, a task which he undertook at the request of the Editorial Committee, of which he was a member. He continued to write the reports of the meetings until 1913, and the records of our meetings have never before or since been so full and accurate. In addition, he had charge of the reviewing section of the *Journal*, and in his quiet way jogged the memories of sluggish reviewers so that authors and publishers had few grounds for complaining of delay in the appearance of book reviews. In 1923–24 he was President of the Society and took as the subject of his address "The Evolutionary History of Renal Surgery and of Temporal Bone Surgery." A conjunction of subjects which denoted his dual rôle as a general and aural surgeon. Firth belonged to the period when aural surgery had not yet become fully allied with laryngology and rhinology to form a speciality.

Firth came to Bristol as House Surgeon to the General Hospital after a distinguished student career at Owens College, Manchester, and University College Hospital, London, where he had been one of Marcus Beck's surgical dressers. He took an exhibition and gold medal in Physiology and Anatomy at London University in 1886, Honours in Anatomy and Materia Medica and Honours in Obstetrics, qualifying M.R.C.S. in 1888, and M.D. London in 1889. In 1893 he obtained the F.R.C.S. England as well as the M.S. London.

In 1896 he was elected Assistant Surgeon to the Bristol General Hospital and Surgeon in 1902. Later he was given charge of the newly formed ear, nose and throat department. He retired from the active staff in 1926, and was made Honorary Consulting Surgeon. During the 1914–18 war he held the rank of Captain R.A.M.C. on the *à la Suite* Staff of the 2nd Southern General Hospital (Territorial) in Bristol. Mr. Lacy Firth was born at Blackburn on January 21st, 1866, the son of Thomas Firth, a Blackburn millowner and Betty Fielden Lacy. In 1908 he married Winifred Mary, daughter of Lewis Edmund Naish (of Bristol) and widow of Henry Ernest Grace (of Bristol). To his widow, we offer our deepest sympathy.

